# Supplementary material for: A Life Dedicated to Climbing and Its Sequelae in the Fingers—A Review of the Literature
Source: Int J Environ Res Public Health. 2022 Dec 19;19(24):17050. doi: 10.3390/ijerph192417050 (PMC9778768; doi:10.3390/ijerph192417050)
Supplement: Supplementary file 1 [file ijerph-19-17050-s001.zip › ijerph-2089760-supplementary.pdf]

Study questionnaire: Risk of Osteoarthritis in Performance Sport climbers

Name: ____________________________________

Surname: ____________________________________

Date of birth: ____________________________________

Height: __________________________________ m

Weight: __________________________________ kg

Profession: ____________________________________

Hand-Dominance: Right / Left

When did you start climbing? Age: _____

How long have you been climbing? (Keyword interruptions) Years:_____

Current training

How often do you train per week? ______________________ h/week

How many hours per week do you climb? ______________ h/week

How many hours per week do you boulder? _____________ h/week

Training behavior over the career

How has the training pattern/week changed during your career?

5 - 10 years of age:_________________ h/week

11 - 20 years of age: _______________ h/week

21 - 30 years of age: _______________ h/week

31 - 40 years of age:________________ h/week

41 - 50 years of age:________________ h/week

51 - 60 years of age:________________ h/week

Best performance

What was your most difficult route redpoint? ______________

What was your most difficult on sight? ______________

What was your most difficult boulder redpoint? _______________

Current climbing performance

route redpoint? ____________________

on sight? ___________________

boulder redpoint? ___________

Climbing level over the career

1.) International elite level (UIAA >XI or >9a), Boulder Fb >8b:

(In years):______ Years of life from to. : _______________________

2.) Elite level (UIAA X+ - XI- or 8b - 8c+), Boulder Fb 7c+ - 8a+:

(In years):______ Years of life from to. : _______________________

3.) Experienced level (UIAA IX- - X or 7b - 8b), Boulder Fb 7a - 7c:

(In years):______ Years of life from to. : _______________________

4.) Intermediate level (UIAA VII- - VIII+ or 6b+ - 7a+), Boulder Fb 6a - 6c+:

(In years): ______ Years of life from to. : _______________________

Diseases

Do you have any disease? □ yes □ no

? ____

Medication

Take medication regularly? □ yes □ no

If yes, which? ____

Finger pain

Have you had pain in your fingers in the last 6 months? □ yes □ no

Which fingers exactly?

____

Have you had pain in your fingers during climbing in the last 6 months? □ yes □ no

Which fingers exactly?

____

Have you had pain in your fingers at rest in the last 6 months? □ yes □ no

Which fingers exactly?

____

Have you ever had an acute injury to the fingers? □ yes □ no

Which fingers exactly?

____

Have you ever had a chronic injury to your fingers? □ yes □ no

Which fingers exactly?

___

How long did you have this pain (in years): ______________________years

Have you ever had surgery on your fingers? □ yes □ no

Which fingers exactly?

___

Which surgery exactly?

___

Morning finger stiffness

Have you had stiffness in your fingers in the last 6 months? □ yes □ no

Which fingers exactly?

___
